# Multiple autoimmune disorders refractory to glucocorticoids after allogeneic hematopoietic stem cell transplantation: a case report and review of the literature

**DOI:** 10.3389/fimmu.2024.1366101

**Published:** 2024-04-19

**Authors:** Linjun Xie, Jingjing Xu, Huiping Xu, Beibei Zhang, Wuqiang Lin, Ting Yang

**Affiliations:** ^1^Department of Hematology, The First Hospital of Putian City, Putian, China; ^2^The School of Clinical Medicine, Fujian Medical University, Fuzhou, China; ^3^Department of Hematology, National Regional Medical Center, Binhai Campus of the First Affiliated Hospital, Fujian Medical University, Fuzhou, China; ^4^Department of Hematology, The First Affiliated Hospital, Fujian Medical University, Fuzhou, China; ^5^Institute of Precision Medicine, Fujian Medical University, Fuzhou, China; ^6^Department of Clinical Nutrition, The First Hospital of Putian City, Putian, China

**Keywords:** allogeneic hematopoietic stem cell transplantation, multiple autoimmune syndrome, autoimmune hepatitis, autoimmune hemolytic anemia, rituximab

## Abstract

We report here the case of a 50-year-old man who was first diagnosed with myelodysplastic syndrome with excess blasts-2 (MDS-EB-2) and underwent allogeneic hematopoietic stem cell transplantation (allo-HSCT) in 2019, resulting in complete remission. However, he was diagnosed in 2021 with several autoimmune disorders, including autoimmune hepatitis (AIH), Hashimoto’s thyroiditis (HT), and autoimmune hemolytic anemia (AIHA). This is referred as multiple autoimmune syndrome (MAS), which is a rare occurrence after allo-HSCT, as previously noted in the literature. Despite being treated with glucocorticoids, cyclosporine A, and other medications, the patient did not fully recover. To address the glucocorticoid-refractory MAS, a four-week course of rituximab (RTX) at a weekly dose of 100mg was administered, which significantly improved the patient’s condition. Thus, this case report underscores the importance of implementing alternative treatments in patients with post-transplant autoimmune diseases, who are glucocorticoid-refractory or glucocorticoid-dependent, and highlights the effectiveness of RTX as second-line therapy.

## 1 Introduction

Allogeneic hematopoietic stem cell transplantation (allo-HSCT) is the treatment of choice for various blood cancers that are not responsive to regular chemotherapy or have a high risk of relapse. However, complications from the transplant can significantly impact the patient’s quality of life and are a major cause of death. Autoimmune diseases (ADs), such as autoimmune hepatitis (AIH), Hashimoto’s thyroiditis (HT), and autoimmune hemolytic anemia (AIHA), are rare complications that can occur after allo-HSCT, events even rarer when all three occur concomitantly. Contributing factors may include delayed Treg recovery, T-cell depletion, and infection-induced autoimmune abnormalities. We are reporting here the first documented case of multiple autoimmune syndrome (MAS) after allo-HSCT in China. The patient did not significantly respond to treatment with glucocorticoids, cyclosporine A (CsA) and other medications, but significantly improved after receiving rituximab (RTX). By reporting this patient’s diagnosis and treatment and undergoing an extensive review of the relevant literature, we aimed to improve our understanding of this rare complication. This study was approved by the Medical Ethics Committee of Fujian Medical University Union Hospital (2022KY167). Informed consent was obtained from all patients.

## 2 Case description

This 50-year-old male was diagnosed with myelodysplastic syndrome with excess blasts-2 (MDS-EB-2; IPSS-R high risk) in January 2019. After 2 weeks of treatment with all-trans retinoic acid and danazol, he received one cycle of bridging chemotherapy with azacytidine combined with CAG (cytarabine, aclacinomycin and G-CSF). The bone marrow blasts proportion decreased from 17% to 7.5%. The patient demonstrated sub-detectable levels of Epstein-Barr virus (EBV), cytomegalovirus (CMV), and hepatitis B virus (HBV) DNA. CMV IgG antibodies were present at 105.9 IU/ml. Serologically, the patient tested negative for HBsAg, HBeAg, and anti-HBe, while testing positive for anti-HBs and anti-HBc antibodies. On April 28, 2019, after pre-treatment with fludarabine (25 mg/m^2^/D on days -12 to -8), cytarabine (2 g/m^2^/D on days -12 to -8), busulfan (3.2 mg/kg/D on days -6 to -4), cyclophosphamide (1 g/m^2^/D on days -6 to -3), and anti-thymocyte globulin (5 mg/kg/D on days -3 to -2), the patient received HLA-matched peripheral blood stem cells from his sister (ABO mismatch A→AB, HBsAg-). The donor’s routine blood tests, liver function, and hepato-biliary ultrasound were all normal, hemolysis and thyroid function were not screened. He was given CsA, methotrexate, and mycophenolate mofetil for prevention of graft-versus-host disease (GvHD). The patient received a single dose of nucleated cells (6.0×10^8^/kg) and CD34+ cells (4.25×10^6^/kg). White blood cells engrafted at Day (D) 14, and platelets at D18. Short tandem repeats (STRs) analysis showed 99.86% chimerism. Upon discharge, the patient received oral CsA and methylprednisolone as well as prophylactic antibiotics. The patient developed CMV infection 3 months post-transplantation, which improved after treatment with ganciclovir. Eight months after transplantation, he developed chronic skin GvHD and lung infection (fungal + bacterial), which improved after anti-infection treatment combined with tacrolimus. The patient was gradually weaned off tacrolimus, CsA, and methylprednisolone during the follow-up period, and the original disease continued to be in remission.

At D619 post-transplantation, the patient’s globulin (GLB) test showed an increase (53 g/L), while total bilirubin (TBil), alanine aminotransferase (ALT), and aspartate aminotransferase (AST) were all within the normal range. On D711, during routine follow-up, the patient was found to be positive for hepatitis B surface antigen (HBsAg), with HBV-DNA levels of 6.52×10^4^ IU/ml and normal liver function. He was treated with the antiviral drug, entecavir (ETV). After 2 months of treatment (+776 days), HBV-DNA was reduced to 1.01×10^4^ IU/ml, but serum ALT and AST rose to 335 U/L and 886 U/L, respectively, while TBil remained normal, and GLB and immunoglobulin G (IgG) levels were high at 75.4 g/L and 57.3 g/L, respectively. Tests for hepatitis A, C, and E, antinuclear antibodies, anti-dsDNA antibodies, and anti-neutrophil cytoplasmic antibodies were negative, and ceruloplasmin was normal. After 10 days of ETV and intravenous liver-protecting treatment, no significant improvement was observed, and the patient was treated with adefovir dipivoxil (ADV) and prednisone (PDN) at 0.5 mg/kg/D. After nearly two weeks of treatment with PDN, dual antiviral therapy (ETV+ADV) and intravenous liver-protecting drugs, liver function returned to normal, and HBV-DNA dropped below the detection limit. The patient continued the dual antiviral therapy and received a maintenance dose of PDN at the reduced dose of 0.25 mg/kg/D, and HBV-DNA remained below the detection limit.

During routine follow-up, the patient’s thyroid-stimulating hormone (TSH) levels were found to be >150 MICRO-IU/L, free triiodothyronine (FT3) was 0.37 pmol/L, and free thyroxine (FT4) was 1.5 pmol/L. Tests revealed HT and hypothyroidism with elevated antithyroid peroxidase antibody (TPOAb) levels at >1300 IU/mL, antithyroglobulin antibody (TgAb) levels of 403.4 IU/mL, and diffuse thyroid lesions. The patient received hormone replacement therapy (levothyroxine), and thyroid function returned to normal.

After 859 days of follow-up, the patient’s TBil levels rose (31.1 mmol/L), while HBsAg was negative, and HBV-DNA was <200 IU/ml. Ursodeoxycholic acid and ademetionine 1,4-butanedisulfonate were used as liver-protecting and jaundice-reducing therapy, but TBil continued to rise, reaching a peak of 118.5 mmol/L. Liver MRI showed iron deposition in the liver parenchyma and spleen, although the patient had received no more than 20 units of suspended red blood cells, the ferritin level was 600.1 ng/ml, and no iron removal therapy was initiated. A diagnosis of liver GvHD was considered, and PDN was increased to 0.5 mg/kg/D, combined with CsA; nevertheless, TBil did not decrease significantly. After 929 days of follow-up, anti-mitochondrial antibodies were negative, while anti-smooth muscle antibodies were positive. Liver biopsy via needle puncture showed moderate lobular and portal hepatitis with bridging fibrosis and mild hemosiderin deposition, with evidence of multinucleated and rosette-like liver cells. Furthermore, chronic inflammation of the bile ducts was observed, with significant plasma cell infiltration and some atrophy, but no loss of the bile duct epithelium. Autoimmune hepatitis (AIH) was considered based on the immunohistochemistry results showing HBsAg (–), CD138 (plasma cell+), CMV (-), D-PAS staining (no alpha-1-antitrypsin granules), iron staining (slight hemosiderin deposition in liver cells), and *in situ* hybridization staining for EBER (-). ADV, CsA, and ademetionine 1,4-butanedisulfonate were stopped, and PDN was increased to 1 mg/kg/D. TBil decreased slightly but did not completely return to normal. PDN was slowly tapered after two weeks and gradually reduced to 0.25 mg/kg/D for maintenance therapy.

At D981, the patient developed fatigue. TBil and IgG were 53.9 mmol/L and 53.2 g/L, respectively, while HGB decreased (54 g/L) and reticulocytes increased (288.9×10^9^/L). The direct antiglobulin test (DAT) was positive, and acid hemolysis test was negative. There was no history of prior infection or suspected drug use. Warm antibody-type autoimmune hemolytic anemia (AIHA) was diagnosed, and A-type washed red blood cells were transfused, with PDN increased again to 1 mg/kg/D.

**Figure 1:**
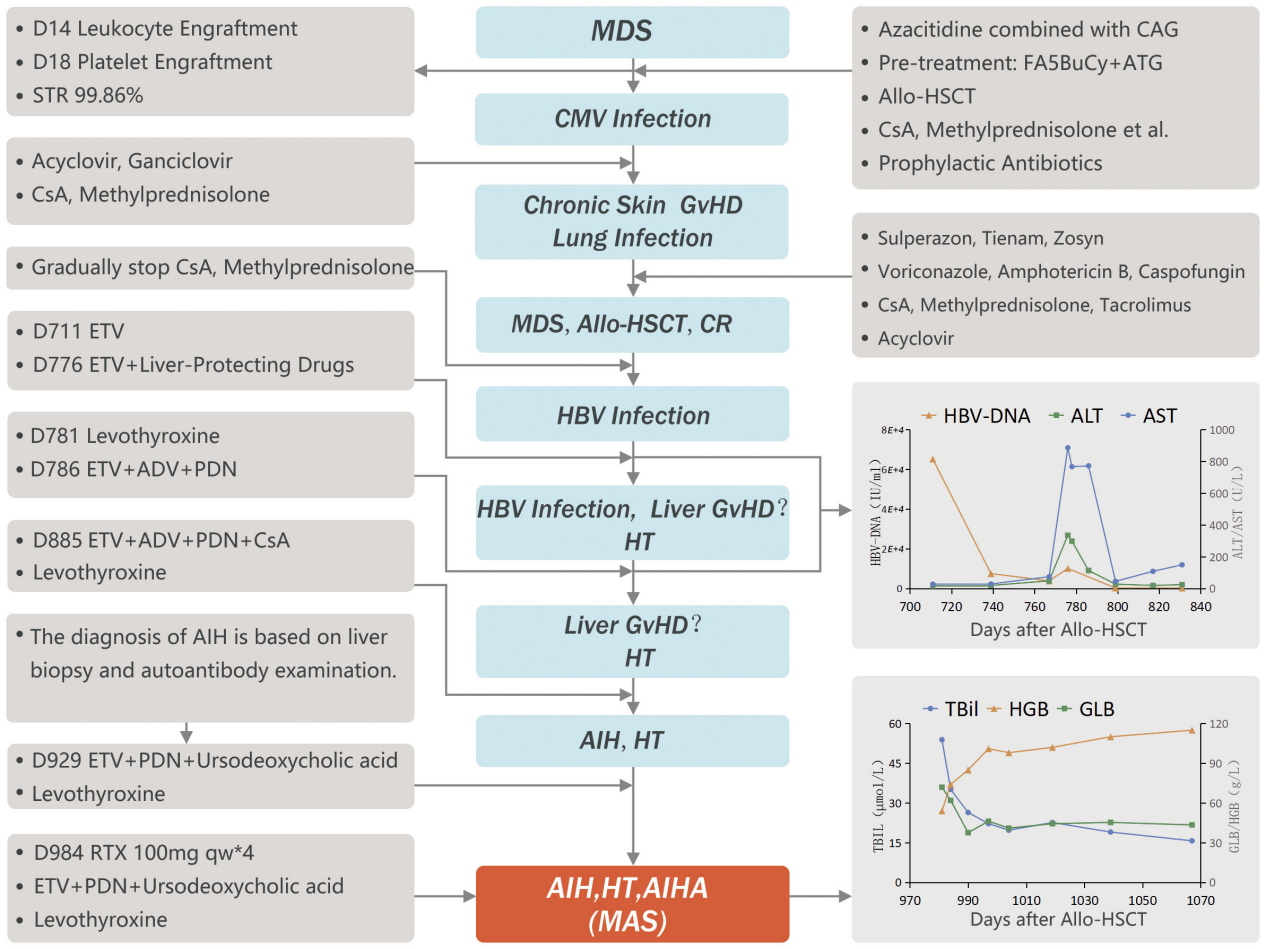
Flowchart.

**Figure 2:**
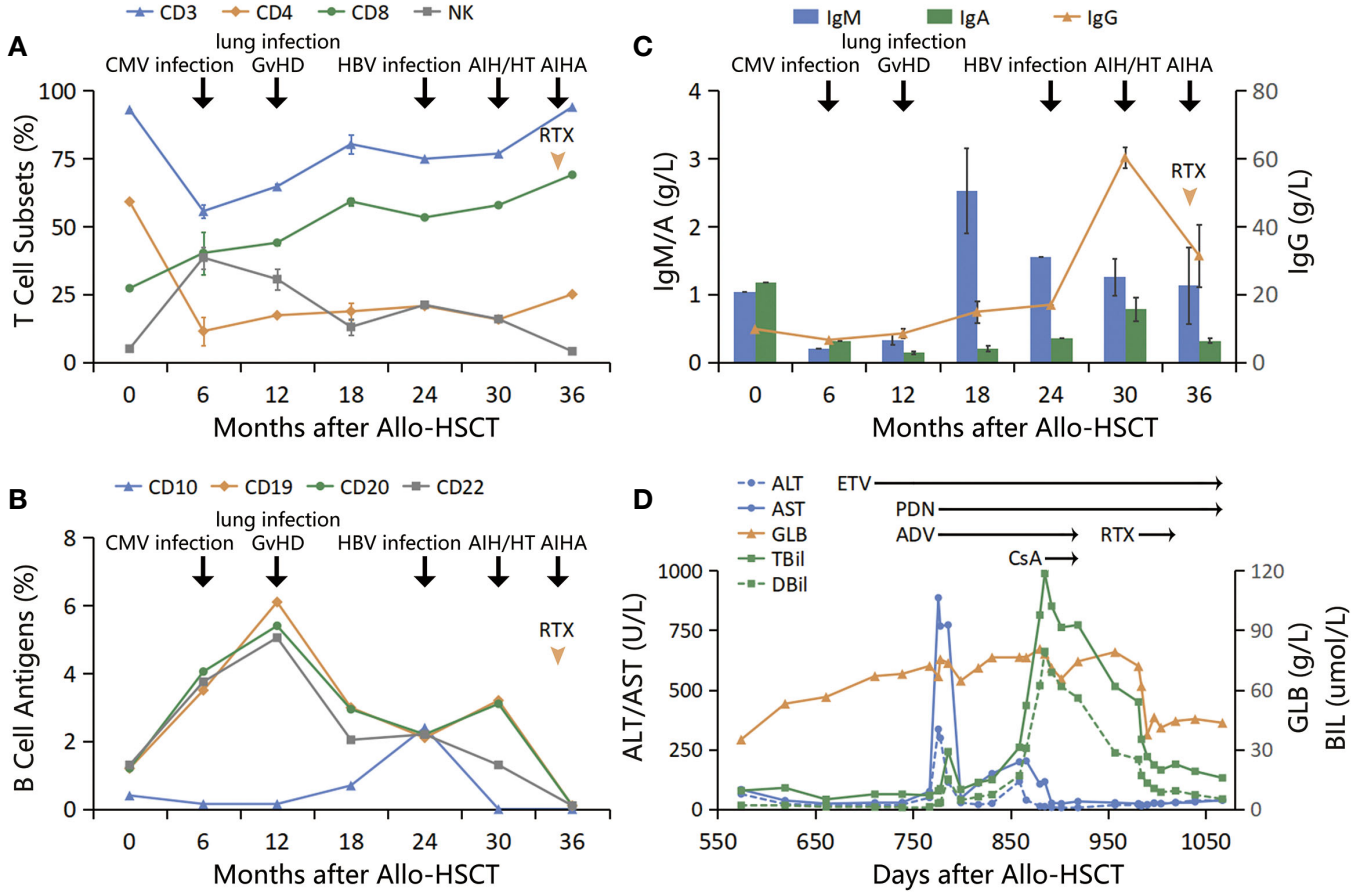
**(A)** T cell subsets: T cells decreased to the lowest level within 6 months post-transplantation, during which CMV infection occurred. Subsequently, CD8+ T cells continued to increase, and GvHD developed, while CD4+ T cells remained at a low level after transplantation; **(B, C)** B cell antigens and immunoglobulins: B cells increased, and immunoglobulins decreased within 1 year after transplantation. Subsequently, B cells decreased while immunoglobulins increased. Both B cells and IgG increased simultaneously after HBV infection; **(D)** Treatment course: GLB increased first, followed by consecutive increases in ALT/AST and TBil to peak values. After anti-HBV treatment and use of PDN and CsA, GLB/IgG did not decrease significantly, TBil did not go back to normal, and new AIHA occurred. After adding RTX, the above indicators improved significantly.

The patient was diagnosed with glucocorticoid-refractory MAS (See **Figure 1** for diagnosis and treatment history). To better control the autoimmune abnormalities, rituximab (RTX) was administered after obtaining informed consent from the patient and his family. The patient’s IgG, TBil, and HGB levels started improving, and PDN was gradually reduced. By D1019, the patient’s IgG levels were 25.5 g/L, TBil was 22.6 mmol/L, HGB was 102 g/L, TSH was 5.548 micro-IU/L, FT3 was 3.06 pmol/L, FT4 was 20.9 pmol/L. PDN was then reduced to a maintenance dose (10 mg/D). At the time of the last follow-up (D 1067), the patient’s IgG levels were 25.4 g/L, TBil was 15.7 mmol/L, HGB was 115 g/L, and T cell subsets and B cell antigens had returned to normal levels (see **Figures 2A–D**).

## 3 Discussion

Autoimmune diseases (ADs) are rare complications of allo-HSCT. Among ADs, the most common is autoimmune cytopenia (AIC), which encompasses AIHA, with an incidence of approximately 4% (1–3). Other complications include AIH and HT, which are even rarer, as the incidence of AIH after allo-HSCT is less than 1% (4), and there is no available large-scale statistical data for the incidence of HT. The concomitant occurrence of at least three types of ADs in the same patient is defined as MAS (5).

Both ADs and GvHD are driven by donor immune reactions, the target in the latter being the host, while ADs affect the donor hematopoietic compartment or non-hematopoietic targets, such as the thyroid, nervous system, skin, liver, and kidneys (6). ADs result from the dysregulation of T and B lymphocytes, aberrant T cell activation and impaired B cell function contribute to the production of autoantibodies, triggering inflammation and tissue damage (7, 8). Within 1 to 2 years after allo-HSCT, naive B-cells typically recover before memory B-cells, but their function remains impaired. CD8+ T cells usually reappear around D 100, while CD4+ T cells, including regulatory T cells (Tregs), may take more than 2 years (9). Tregs can inhibit immune reactions and maintain self-immune tolerance. If Treg recovery is delayed or severe T-cell depletion occurs, the proliferation of self-reactive lymphocytes that recover first may lead to ADs (10). Preconditioning regimens containing ATG or alemtuzumab can cause severe T-cell depletion, and the need for intensified immunosuppressive therapy for chronic GvHD may also delay immune recovery. Infection could induce autoimmune abnormalities, which have been identified as risk factors for ADs after allo-HSCT in multiple studies (8, 10).

In our case, the patient’s B cells increased early post-transplantation, while immunoglobulins decreased, which may have been due to the naive B-cells having deficient immune function and entering the peripheral blood first, followed by the release of mature B-cells and gradual reconstitution of humoral immune function. The patient subsequently developed multiple infections with pathogens such as CMV, fungi, and HBV, which may have induced B-cell proliferation and hyperfunction. Furthermore, the patient underwent a pre-transplant conditioning regimen containing ATG and received prolonged immunosuppressive therapy for chronic GvHD, which might be associated with the long-term maintenance of low levels of CD4+ T cells. These factors could potentially contribute collectively to the subsequent development of multiple ADs.

This case was initially diagnosed as chronic hepatitis B and received ETV antiviral treatment. Despite the patient’s decreasing HBV-DNA levels, the serum aminotransferase levels continued to rise. The patient then showed improvement after combined treatment with PDN, indicating that a simple chronic hepatitis B was not responsible for the patient’s condition. Ultimately, based on the simplified diagnostic criteria for AIH (11), the patient exhibited a positive SMA antibody (1 point), negative HBsAg in both blood and liver tissue (2 points), IgG levels greater than 1.10 times the upper normal limit (2 points), and liver histology consistent with typical AIH (2 points), resulting in a total score of 7 points. Therefore, the data supported a diagnosis of AIH.

Regarding the treatment of ADs after allo-HSCT, there is currently a lack of standardized procedures in the literature, and treatment is often guided by experience from the treating physicians. The American Association of Liver Diseases recommends PDN alone or in combination with azathioprine as the first-line treatment for AIH (12), but there is limited data for the treatment of AIH after allo-HSCT, and some reports have suggested that glucocorticoid therapy is ineffective (13–16). In one case, remission was achieved using RTX, and a follow-up liver biopsy showed complete disappearance of CD20+B-cells and CD138+ plasma cell infiltration, with maintenance of biochemical remission after discontinuation of glucocorticoid therapy (16). Burak et al. (17) reported successful treatment with RTX in 6 AIH patients who failed to respond to PDN and azathioprine treatment. Similar treatment responses have been observed in allo-HSCT-related AIHA, with the response to corticosteroid treatment generally being modest (18, 19). A study by M. Wang et al. (1) showed that 74% of patients with post-transplant AIHA required second-line or even third- or fourth-line treatment, but 46% achieved remission with RTX combined with corticosteroids or other immunosuppressive agents. Research in China also confirmed the effectiveness of RTX in treating patients with post-transplant AIHA who failed conventional treatment (2, 20). In our case, TBil did not return to normal levels despite the use of PDN and CsA. IgG levels remained elevated, and onset of AIHA occurred during treatment, indicating that abnormal immunity was not under control. Consequently, corticosteroid resistance or refractory ADs was considered. After a literature review, we used RTX in combination with PDN to control both AIH and AIHA. The aforementioned indicators showed significant improvement. However, the long-term efficacy of protocol needs further evaluation. While recognizing the effectiveness of RTX, it’s crucial to address potential challenges, including the risk of HBV reactivation. Therefore, continuous antiviral therapy and vigilant monitoring of liver function and HBV DNA levels are imperative when using RTX as a second-line treatment for glucocorticoid-refractory ADs.

In conclusion, ADs are rare late complications of allo-HSCT, resulting in poor prognosis. With this report of the first case of MAS after allo-HSCT and our extensive literature review, our goal was to increase awareness and facilitate early recognition to implement aggressive treatment. Given the often-observed poor response to frontline therapy, early combination treatment with RTX may provide significant benefits to such patients, while further studies would be needed to assess the efficacy of novel agents.

## Data availability statement

The original contributions presented in the study are included in the article/supplementary material. Further inquiries can be directed to the corresponding author.

## Ethics statement

The studies involving humans were approved by the Ethics Committee of Union Hospital Affiliated to Fujian Medical University. The studies were conducted in accordance with the local legislation and institutional requirements. The participants provided their written informed consent to participate in this study. Written informed consent was obtained from the individual(s) for the publication of any potentially identifiable images or data included in this article.

## Author contributions

LX: Writing – original draft, Conceptualization. JX: Writing – original draft. HX: Data curation, Visualization, Writing – review & editing. BZ: Data curation, Formal analysis, Writing – review & editing. WL: Supervision, Writing – review & editing. TY: Validation, Writing – review & editing.

## References

[1] Wang M Wang W Abeywardane A Adikarama M McLornan D Raj K. Autoimmune hemolytic anemia after allogeneic hematopoietic stem cell transplantation: analysis of 533 adult patients who underwent transplantation at King's College Hospital. Biol Blood Marrow Transplant. (2015) 21:60–6. doi: 10.1016/j.bbmt.2014.09.009 25262883

[2] Yang Z Wu B Zhou Y Wang W Chen S Sun A. Clinical and serological characterization of autoimmune hemolytic anemia after allogeneic hematopoietic stem cell transplantation. Chin Med J. (2014) 127:1235–8. doi: 10.3760/cma.j.issn.0366-6999.20132823 24709172

[3] Sanz J Arriaga F Montesinos P Orti G Lorenzo I Cantero S. Autoimmune hemolytic anemia following allogeneic hematopoietic stem cell transplantation in adult patients. Bone marrow Transplant. (2007) 39:555–61. doi: 10.1038/sj.bmt.1705641 17351645

[4] Koyama D Ito M Kamoshita S Kubota N Yokohata E Goto T. Autoimmune-like hepatitis following allogeneic hematopietic stem cell transplantation: different manifestation from hepatic graft-versus-host disease. Blood. (2011) 118:4550. doi: 10.1182/blood.V118.21.4550.4550

[5] Cojocaru M Cojocaru IM Silosi I. Multiple autoimmune syndrome. Maedica. (2010) 5:132. doi: 10.1016/S0248-8663(05)81166-X 21977137 PMC3150011

[6] Buxbaum NP Pavletic SZ. Autoimmunity following allogeneic hematopoietic stem cell transplantation. Front Immunol. (2020) 11:2017. doi: 10.3389/fimmu.2020.02017 32983144 PMC7479824

[7] Meffre E O'Connor KC. Impaired B-cell tolerance checkpoints promote the development of autoimmune diseases and pathogenic autoantibodies. Immunol Rev. (2019) 292:90–101. doi: 10.1186/s13044-018-0046-9 31721234 PMC9145185

[8] Rydzewska M Jaromin M Pasierowska IE Stożek K Bossowski A. Role of the T and B lymphocytes in pathogenesis of autoimmune thyroid diseases. Thyroid Res. (2018) 11:1–11. doi: 10.1111/imr.12821 29449887 PMC5812228

[9] Ogonek J Kralj Juric M Ghimire S Varanasi PR Holler E Greinix H. Immune reconstitution after allogeneic hematopoietic stem cell transplantation. Front Immunol. (2016) 7:507. doi: 10.3389/fimmu.2016.00507 27909435 PMC5112259

[10] Li Z Rubinstein SM Thota R Savani M Brissot E Shaw BE. Immune-mediated complications after hematopoietic stem cell transplantation. Biol Blood Marrow Transplant. (2016) 22:1368–75. doi: 10.1016/j.bbmt.2016.04.005 27095688

[11] Hennes EM Zeniya M Czaja AJ Parés A Dalekos GN Krawitt EL. Simplified criteria for the diagnosis of autoimmune hepatitis. Hepatology. (2008) 48:169–76. doi: 10.1002/hep.22322 18537184

[12] Mack CL Adams D Assis DN Kerkar N Manns MP Mayo MJ. Diagnosis and management of autoimmune hepatitis in adults and children: 2019 practice guidance and guidelines from the American Association for the study of liver diseases. Hepatology. (2020) 72:671–722. doi: 10.1002/hep.31065 31863477

[13] Ogose T Watanabe T Suzuya H Kaneko M Onishi T Watanabe H. Autoimmune hepatitis following allogeneic PBSCT from an HLA-matched sibling. Bone marrow Transplant. (2003) 31:829–32. doi: 10.1038/sj.bmt.1703923 12732893

[14] Granito A Stanzani M Muratori L Bogdanos DP Muratori P Pappas G. LKM1-positive type 2 autoimmune hepatitis following allogenic hematopoietic stem-cell transplantation. Off J Am Coll Gastroenterology| ACG. (2008) 103:1313–4. doi: 10.1111/j.1572-0241.2007.01782_7.x 18477361

[15] Mori M Tabata S Hashimoto H Inoue D Kimura T Shimoji S. Successful living donor liver transplantation for severe hepatic GVHD histologically resembling autoimmune hepatitis after bone marrow transplantation from the same sibling donor. Transplant Int. (2010) 23:e1–4. doi: 10.1111/j.1432-2277.2009.01028.x 20028495

[16] Narita A Muramatsu H Takahashi Y Sakaguchi H Doisaki S Nishio N. Autoimmune-like hepatitis following unrelated BMT successfully treated with rituximab. Bone marrow Transplant. (2012) 47:600–2. doi: 10.1038/bmt.2011.124 21666737

[17] Burak KW Swain MG Santodomino-Garzon T Lee SS Urbanski SJ Aspinall AI. Rituximab for the treatment of patients with autoimmune hepatitis who are refractory or intolerant to standard therapy. Can J Gastroenterol. (2013) 27:273–80. doi: 10.1155/2013/512624 PMC3735730 23712302

[18] Daikeler T Labopin M Ruggeri A Crotta A Abinun M Hussein AA. New autoimmune diseases after cord blood transplantation: a retrospective study of EUROCORD and the Autoimmune Disease Working Party of the European Group for Blood and Marrow Transplantation. Blood J Am Soc Hematol. (2013) 121:1059–64. doi: 10.1182/blood-2012-07-445965 23247725

[19] Rovira J Cid J Gutiérrez-García G Pereira A Fernández-Avilés F Rosiñol L. Fatal immune hemolytic anemia following allogeneic stem cell transplantation: report of 2 cases and review of literature. Transfusion Med Rev. (2013) 27:166–70. doi: 10.1016/j.tmrv.2013.02.004 23562007

[20] Wang C Xue SL Li Z Bao XB Chu XL Han R. Autoimmune hemolytic anemia after allogeneic hematopoietic stem cell transplantation: report of one case and review of literature. J Leuk Lymphoma. (2018) 27:228–233,237. doi: 10.3760/cma.j.issn.1009-9921.2018.04.009

